# Is the Family Health Center Professionals’ Knowledge Level About Child Abuse and Neglect Sufficent? A Comprehensive Field Study

**DOI:** 10.3390/children12010088

**Published:** 2025-01-14

**Authors:** Serdar Deniz, Muhammet Bayraktar, Tufan Nayir, Elçin Balcı, Feyza İnceoğlu

**Affiliations:** 1 Department of Public Health, Faculty of Medicine, Malatya Turgut Ozal University, 44210 Malatya, Türkiye; serdar.deniz@ozal.edu.tr; 2 Department of Public Health, Faculty of Medicine, Nigde Omer Halisdemir University, 51240 Nigde, Türkiye; muhammetbayraktar@ohu.edu.tr; 3 Ministry of Health, 06800 Ankara, Türkiye; tufannayir@gmail.com; 4 Department of Public Health, Faculty of Medicine, Erciyes University, 38030 Kayseri, Türkiye; ebalci@erciyes.edu.tr; 5 Department of Biostatistics, Faculty of Medicine, Malatya Turgut Ozal University, 44210 Malatya, Türkiye

**Keywords:** child, abuse, neglect, family health professional, physician

## Abstract

Background: Child abuse and neglect bring lots of undesirable consequences for the future of children and societies with it. It is expected that health service providers have sufficient knowledge about that subject in order to determine abuse and neglect. Materials and methods: In this study, a 67-item scale with a Cronbach’s alpha value of 0.92 was used. A score of more than 3 on the scale meant that the score was at a sufficient level. The universe of this descriptive study is health professionals working in 512 family health units in Mersin, Türkiye. Interviews were conducted with 97.26% (*n* = 249) of family physicians and 98.04% (*n* = 251) of family health workers working in the 256 units included in the sample. Results: Since the overall scale score of the study group was above 3, it (3.77 ± 0.36) was determined that the average score of the participants was sufficient. 46.2% (*n* = 231) of the group did not receive training on child neglect and abuse during their education, and 45.2% (*n* = 226) during their work. 95.8% stated that they paid attention to signs of abuse and neglect in children brought for examination and follow-up, while 83.4% stated that they had not encountered any cases in the last year. 42% (*n* = 210) of the group thought that the relevant authorities would not take the necessary action even if they observed signs of child neglect and abuse, while 37.4% (*n* = 187) stated that they did not know when and how to report. Conclusions: Although the findings of the study suggest that the group’s level of knowledge is sufficient, it is of great importance to increase their knowledge about reporting processes and to encourage them in this regard.

## 1. Introduction

Violence manifests itself in physical, sexual, and psychological dimensions as deprivation and/or neglect. These dimensions are often associated with child maltreatment. Child maltreatment is any kind of physical and emotional abuse, sexual abuse, neglect, or exploitation that may be harmful to the child’s health, life, development, and dignity [1,2]. There are some cases that can be considered as risk factors for child maltreatment and are caused by the child, their parents, those who are responsible for their care, and the community. Being an unwanted child, being under 4 years old or adolescent, and having special needs or disabilities are considered risk factors for children. Having had attachment problems in the neonatal period, having been ill-treated as a child, having non-realistic expectations, using alcohol and drugs, experiencing financial problems, and having committed a crime in the past are risk factors for parents and those responsible for the child’s care [3]. Some of the social risk factors in child abuse or neglect are; gender discrimination, social inadequacy, poverty, inadequate policies on child abuse and neglect, cultural norms that encourage violence and disrupt child-parent relations, as well as inadequacies in health and education policies [1,2].

According to WHO data; one-fourth of adults reported having been physically abused, while one-fifth of women and one in thirteen of the men reported having been sexually abused during their childhood [2,4]. Improper approaches to children and maltreatment can adversely affect the future of a country and create negative economic and social consequences. Programs created with a multisectoral approach for parents can prevent maltreatment and reduce the risk of recurrence. As even some parents state that they use sorts of violence to discipline their children [4,5].

Child abuse includes physical, sexual, and emotional abuse as well as neglect [4]. Physical abuse should come to mind when causes of bruisings, burns, cutting tools traumas, poisoning, asphyxia, bite marks, abrasions, and suffocation cannot be explained sufficiently by the parents [6,7,8,9,10,11]. The most common finding of physical abuse is skin lesions [6,11]. Emotional abuse should also be considered in children who are deprived of attention, love, and care. In addition to physical and emotional abuse; children who exhibit unexpected sexual behavior and who have signs of genital bleeding and sexually transmitted diseases should be evaluated in terms of sexual abuse. Nonspecific findings such as sleep disturbance, enuresis, and encopresis can also accompany the first presentation of sexual abuse [10]. Sexual abuse also has a wide network of findings, including intra-oral lesions, such as other types of abuse and may result in memory loss, eating disorders, anger, depression, and aggression [7,8,10]. It is a sign of the child is being neglected when there is deprivation or lack in the child’s basic requirements such as nutrition, cleanliness, education, and health. The neglected child exhibits aggression in the future and has various emotional problems along with learning and self-confidence problems [7,9].

Health professionals have ethical and legal responsibilities on this subject. Family health centers are the closest and easiest to reach health facilities and they also take the role of the gatekeepers of the health services system [12]. As one of the most important studies conducted out in these centers is the monitoring of infants, children, and adolescents [13], family health centers can also be seen as gatekeepers to prevent child abuse and neglect. In family health centers, family physicians and family health workers who work together as family health staff perform their studies according to the relevant legislation [12]. Until the baby completed her first year, she is assessed at least nine times. Twice in the hospital where she was born and seven more times at the family medicine unit on the dates set according to the date of her birth. Findings are recorded in the family medicine information system. Similarly, childhood period follow-up studies are also carried out [12,13]. One of the points to be taken into consideration during the follow-ups is to observe and question whether babies, children, or adolescents are exposed to neglect and ill-treatment. It is expected from a family physician or family health worker who performs follow-ups, to take the necessary actions and to correctly recognize the signs in case the minor patient is exposed to physical maltreatment and in case his psychosocial development is adversely affected. However, previous researches hint at the lack of knowledge and awareness of primary healthcare workers on the signs and symptoms of child abuse and neglect. For this reason, the notification process and legal issues related to child abuse should be included in their education programs [7,14,15,16,17].

The first paragraph of Article 6 of the Child Protection Law (CPL) states that “Judicial and administrative authorities, law enforcement officers, health and education institutions, and non-governmental organizations are obliged to report a child in need of protection to the Social Services and Child Protection Agency”. A child in need of protection may be a child who is the victim of a crime, or a child who is neglected or abused, whose physical, mental, moral, social and emotional development and personal safety are at risk. If one of the persons listed in the first paragraph of Article 6 of the CPL encounters such a child and does not fulfill the reporting obligation, unless the act violates items 278, 279 and 280 of the Turkish Penal Code (TPC); the crime of negligent abuse of duty regulated in the second paragraph of Article 257 of the TPC will occur for public officials [18,19,20].

A special reporting obligation is also foreseen exclusively for healthcare professionals in Article 280 of the TPC. This article imposes an obligation to report crimes on healthcare professionals who encounter indications that a crime has been committed while performing their duties. The term healthcare professional refers to physicians, dentists, pharmacists, midwives, nurses and other persons providing healthcare services (TPC item 280/2). In order for this crime to occur, the crime must be learned by the healthcare professional in connection with the performance of their duties. In other words, the healthcare professional must encounter the indication constituting a crime while performing their duties [20,21].

To eliminate the lack of knowledge and awareness through interventions such as in-service training as required; it is necessary to determine in which group, with what variables, and to what extent the deficiency is affected. Again, it is imperative to know in which topics there are knowledge gaps in the child abuse and neglect [CAaN] context to plan necessary actions to fulfill them. To ensure maximum benefit from any training, it is necessary that the people who will receive the training are aware of their needs and are willing to be trained. So information about the interests of health workers on the subject is also collected in the scope of this study.

The aim of the study is to measure the knowledge levels and approaches of primary health care workers about child neglect and abuse and to determine the associated factors.

## 2. Materials and Methods

**Figure 1:**
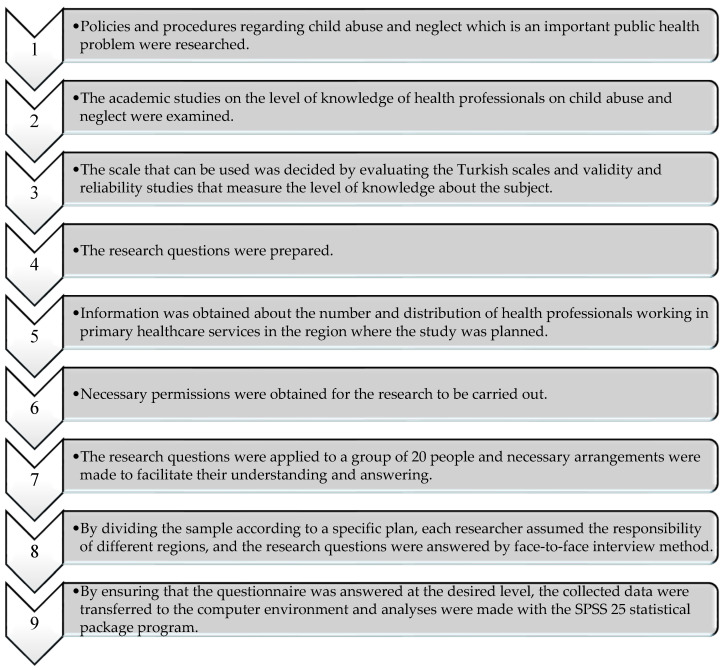
Research Flow Chart.

This cross-sectional study was conducted through the following steps is given Figure 1.

At the time the study was conducted, Mersin, Türkiye’s 11th largest city by population, had a population of 1,793,931 [22]. According to health policies in Türkiye, planning was made to have approximately 1 family doctor per 3500 population. For this reason, there were 512 family medicine units in Mersin (1 family doctor and 1 family health worker in each unit).

The population of this descriptive study is composed of family physicians and family health workers working in 512 family health units in Mersin. The frequency of defining symptoms and risks of child abuse and neglect in family health units was taken as 50% to reach the maximum sample size, with an error rate of 5% and a confidence interval of 95%. The Epi Info program has calculated the minimum sample size with these parameters as 219 units. The sample size increased by 15% with the aim of reaching the family physician and family health workers working in 256 family health units due to the probability of their not accepting to participate in the study and not being able to reach the whole sample due to being on leave, etc. Each family medicine unit in a city has a number. These numbers start from 1 and continue until the last family medicine unit number. During the study period, there were 512 family medicine units (1 family physician and 1 family health worker in each unit) in Mersin. As a result of the calculation, after all units were ranked from 1 to 512, odd numbered units (1, 3, 5, 7, …, 507, 509, 511) were selected and 256 units (256 family physicians and 256 family health workers) were included in the sample. Required permissions were obtained from Erciyes University Clinical Research Ethics Committee on 21 April 2017 and from Türkiye Public Health Agency on 31 July 2017. The questionnaire consisting of participant information form of 20 questions and a scale of 67 questions was applied using face-to-face interview techniques to the volunteers from the sample. The field studies of research started on 18 December 2017 and were completed on 30 April 2018. Because the physicians’ and family health workers’ included in the sample were absent for several reasons like missing, being on leave, or having a rest for being ill; 97.26% (*n* = 249) of family physicians and 98.04% of family health workers; consisting of midwives, nurses, public health technicians, emergency medical technicians; were interviewed. Informed consent was obtained from the participants and included in the study.

### 2.1. Data Collection Tools

#### 2.1.1. Personal Information Form

The socio-demographic data form was applied to the participants.

#### 2.1.2. The Diagnosis Scale of the Risks and Symptoms of Child Abuse and Neglect

The scale used in our study: The Diagnosis Scale of The Risks and Symptoms of Child Abuse and Neglect. In the scale used; there are items that measure the level of knowledge about Physical Symptoms of Child Abuse (PSoCA, 19 items), Symptoms of Child Neglect (SoCN, 7 items), Behavioral Symptoms of Child Abuse and Neglect (BSoCAaN, 15 items), Characteristics of Parents Prone to Child Abuse and Neglect (CPPtCAaN, 12 items), Characteristics of Children Prone to Abuse and Neglect (CCPtAaN, 6 items), Family Characteristics in Child Abuse and Neglect (FCiCAaN, 8 items) [23].

The validity and reliability study of the scale consisting of 67 questions was performed by Uysal. The Cronbach’s alpha value of it was 0.92. The point values of 46 of the 67 questions in the scale are as; Very accurate = 5, Quite true = 4, Unsure = 3, Not true = 2, Not true at all = 1 point. For the remaining 21 questions (3, 5, 8, 10, 12, 14, 16, 27, 28, 30, 32, 34, 41, 42, 46, 49, 54, 56, 59, 61, 63) the point values are the opposite of the other 46 questions (Very accurate = 1, Quite true = 2, Unsure = 3, Not true = 4, Not true at all = 5). The following formula was applied while calculating the average score for the scale and its subcategories: Score = (Total of points in the related category/Number of questions in the related category). In this way, the highest average score will be five and the lowest score will be one. Scores close to five indicate better knowledge of the subject, while scores close to one indicate the opposite. That can also be interpreted as scores above three mean adequate knowledge of the topic and below three mean non-adequate knowledge of the topic [23].

### 2.2. Statistical Analysis

The conformity of the data to the normal distribution was determined by the Kolmogorov-Smirnov test. Means were presented with their standard deviations, numbers were presented with their percentages. The differences of two means were evaluated by Student’s *t*-test, and the differences of more than two means were evaluated by one way analysis of variance. A multivariate linear regression model was used to build a prediction model with the variables included in the study. The variables were checked for multivariate normal distribution, multicollinearity, and autocorrelation between error coefficents. Variables were included in the model with the Enter method. Statistical significance level was determined as *p* < 0.05.

## 3. Results

The mean age of our study group was 42.68 ± 8.90, 36.6% (*n* = 183) are males, and 49.8% (*n* = 249) are doctors. 87.8% (*n* = 439) of the group were married during the study and 89.0% (*n* = 445) had at least one child. The descriptive characteristics of the participants are presented in Table 1.

Table 2 presents the distribution of participants’ responses to questions about the training they took and their past experiences with child neglect and abuse cases. 46.2% (*n* = 231) of the group had not received training on child neglect and abuse during their professional life and 45.2% (*n* = 226) during their education.

The overall scale score of the study group was calculated as 3.77 ± 0.36 (min. 2.91–max. 4.70). The mean scores for subcategories were found as follows: PSoCA = 4.02 ± 0.42 (min. 2.95–max. 5.00), SoCN = 3.87 ± 0.60 (min. 2.14–max. 5.00), BSoCAaN = 3.79 ± 0.39 (min. 2.53–max. 4.87), CPPtCAaN = 3.50 ± 0.49 (min. 2.08–max. 5.00), CCPtAaN = 3.32 ± 0.50 (min. 1.83–max. 5.00), FCiCAaN = 3.81 ± 0.58 (min. 2.50–max. 5.00) (Table 3). The most frequent (62.8%) correct response was seen in the item “Vaginal and rectal bleeding can be seen as a result of sexual abuse” (Table 3).

Table 4 shows the comparison of the scale scores in terms of gender (1st line), marital status (2nd line), having children status (3rd line), age groups (4th line) and education status (5th line). The mean value for male participants was higher in all categories compared to the women’s average, but it was found that the only category in which this difference was significant was CPPtCAaN (*p* < 0.05). The mean scale and sub-category scores of the married and unmarried ones were similar (*p* > 0.05). The mean SoCN scores of participants who have a child were higher than those who have not (*p* < 0.05). The only subscale where a statistically important difference was observed between those with associate degrees and higher education levels versus those with high school and lower education levels is CPPtCAaN (*p* < 0.05).

Table 5 shows the comparison of the scale scores in terms of, occupation (line 1), the status of receiving child neglect and abuse training during education (2nd line) and throughout professional life (3rd line), the status of paying attention to the signs of abuse and neglect in infants and children during examination and follow-up (4th line), the status of encountering child neglect or abuse in working life (5th line), the status of wanting to attend a training on child neglect and abuse (line 6) and total working time in occupation (7th line). While the mean scores of SoCN, CCPtAaN, and FCiCAaN for family physicians and family health workers were similar (*p* > 0.05), family physicians had higher scores in the overall scale average, PSoCA, BSoCAaN, and CPPtCAaN (*p* <0.05).

The overall scale score and the sub-category scores of all age groups were similar in terms of age groups (*p* > 0.05). It was determined that the only category where the total working time in the profession has an effect was the SoCN. The difference in this category was the result of the difference between the employees who worked for the longest time and the shortest time (*p* < 0.05). Total working time in the profession had any effect on neither the overall scale score nor any other subcategories (*p* > 0.05) (Table 5).

Table 6 shows that distribution of the reason why physicians who observe evidence of child abuse and/or neglect do not report this to the relevant authorities according to variables. 89% of the group (*n* = 445) had not reported child abuse and neglect. The most common reason given was that they thought the relevant authorities would not do what was necessary. Another common response was that they did not know how and when to report. A desire not to get involved in an unpleasant topic was also a frequently reported reason.

Table 7 shows that distribution of the topics to be covered in a training on child abuse and neglect according to variables. 94% (*n* = 470) of the participants answered the question about the topics they recommended to be covered in training on child abuse and neglect. The most requested topic among those who answered the question (83.8%) was “Information on what can be done in cases of detection of child abuse and/or neglect”. Then, “Information on the experiences of children who have been reported to the authorities for abuse and/or neglect after this process” (74%), “Information on legal regulations regarding the reporting of child abuse and/or neglect” (72.6%) and “Information about the signs and symptoms of child abuse and neglect” (72.6%) were specified.

The analysis results of the multivariate linear regression model in which The Diagnosis Scale of The Risks and Symptoms of Child Abuse and Neglect scores were taken as the dependent variable are given in Table 8. 4.6% of the change in the Diagnosis Scale of the Risks and Symptoms of Child Abuse and Neglect score is explained by their position at the family health center, their total working time, and whether or not they received Diagnosis Scale of the Risks and Symptoms of Child Abuse and Neglect training (R^2^ = 0.046). The Diagnosis Scale of The Risks and Symptoms of Child Abuse and Neglect score is higher in family health workers than family physicians (β_1_ = −0.147; *p* = 0.001). Awareness of The Diagnosis Scale of The Risks and Symptoms of Child Abuse and Neglect decreases as the duration of employment increases (β_1_ = −0.047; *p* = 0.031). The Diagnosis Scale of The Risks and Symptoms of Child Abuse and Neglect scores decreased as the duration of employment increased in those who received training on the subject (β_1_ = −0.089; *p* = 0.001). The most important factors for awareness of The Diagnosis Scale of The Risks and Symptoms of Child Abuse and Neglect were working as a family physician, receiving training on the subject, and being in the first years of working in the profession.

## 4. Discussion

In our study, the mean age of the study group was 42.68 ± 8.90 years. More than half of the group consisted of female health workers. However, more than 80% of them were married and had at least one child. The previous studies using the same scale as our study have been found to have similar findings socio-demographically [23,24,25,26,27].

Those who were paying attention to the findings of child abuse and neglect were 95.8% of the group, and in a study performed in 2012 a very close result was obtained [25]. 27.4% of the study group experienced child neglect and abuse cases during their working life. In other studies conducted with the scale used in our study, it was observed that more than 30% of health personnel encountered cases of child abuse. Those who took training about child abuse and neglect pre-graduation are 53.8% of the group and 54.8% took training on the topic post-graduation. It was found that the status of getting an education before and after graduation was 26.7–70.6% and 6.1–83.8% in similar studies, respectively. It was observed that the group that received the highest education after graduation was identified in the research by Türker [27]. As in our research, in the other studies carried out in Türkiye ratio of those who want to participate in education or information activities related to child abuse and neglect is higher than 80% [23,24,25,26,27]. In another study, it was determined that 10% of the participants encountered at least one CAaN case in the last year [28].

In the evaluation made in terms of mean scores of the whole group; Demir’s research in physicians (3.86 ± 0.33) and Kocaer’s research in physicians and nurses (3.65 ± 0.33), although they were performed 5 and 11 years ago, compared to the general scale mean score of our study (3.77 ± 0.36) similar results were obtained. In the same studies; PSoCA (respectively 4.05 ± 0.38 and 3.86 ± 0.38, our study; 4.02 ± 0.42), SoCN (4.01 ± 0.51 and 3.86 ± 0.48, our study 3.87 ± 0.60), BSoCAaN (3.88 ± 0.41 and 3.80 ± 0.38, our study; 3.79 ± 0.39), CPPtCAaN (3.64 ± 0.45 and 3.46 ± 0.47, our study; 3.50 ± 0.49), CCPtAaN (3.45 ± 0.55 and 3.23 ± 0.62, our study; 3.32 ± 0.50), and FCiCAaN (3.86 ± 0.51 and 3.74 ± 0.52, our study; 3.81 ± 0.58) results for the subcategories are also very close to the results of our research [24,25].

According to our findings, the item with the lowest mean score (1.99 ± 0.98) was “Children who are subjected to abuse and neglect are overreacting against painful stimuli and traumas”. From the BSoCAaN subcategory. According to the results of the researches conducted by Uysal and Demir, the same item also had an average score [23,25].

In the general scale average; there are no point differences related to mean age, gender, marital status, child status, and total working time. Gender in research of Gölge et al. [26], age, gender, marital status and total working time in research of Türker [27], age, marital status, having a child and working time in research of Kocaer [24], having a child and working time at a family health center in Demir’s [25] research were found to have no affects on general scale point average. Demir’s [25] research showed that the general scale score decreased with age and it was also observed that the average score of the men was higher than the women. That contradicts the data in the literature that show women have a higher level of knowledge on child abuse and neglect topic than men [29].

In other research examined; in the study of Arıkan et al. [30]; age group, marital status, having a child, and total working time did not have an effect on knowing the definition of child neglect and abuse. In a study conducted only on physicians, it was found that the CAaN knowledge points were higher in older people, women, married people, and those with more total working time [31].

The general scale mean scores of the family physicians were higher than those of the family health workers. Similarly, the mean score of the physicians was higher than the non-physician participants in the studies conducted by Türker [27] and Kocaer [24]. In the study by Gölge et al. [26] the mean score of the physicians and the non-physicians are found to be similar. In a study conducted on physicians [pediatrician, pediatric assistant, and general practitioner], it was determined that the CAaN knowledge scores of the assistant, general practitioner, and specialist physicians were also similar [31].

Previously having diagnosed child abuse and neglect and paying attention to CAaN findings during examinations and follow-ups have no effect on scale and subcategory point averages. Demir’s [25] research in this regard has been supporting our findings. In the other study, it was found that the mean scores on the subject were higher than those who had previously encountered CAaN cases [31].

When the results of the research using the same scale in previous years are examined; the tendency of the answers to the scale questions and the scores obtained do not show a significant change although there are long periods between the studies. According to these results, it can be thought that due to insufficient research on the subject of CAaN, no interventions were made for the cause, and the deficiencies in the subject of CAaN continue from the results of different studies. While the physicians and other health professionals in family health centers are one of the professional groups with a high probability of getting across to an abused or neglected child, our study revealed that their knowledge levels about child abuse and neglect should be improved. Tuğut and Daşlı’s [32] findings also hint that the family health center professionals’ awareness of child abuse and neglect was not sufficient. As reported by a recent study it is possible to increase the sensitivity and awareness and professional healthcare workers with pieces of training on violence and violence education to help improve healthcare professionals’ thoughts and attitudes on the subject [33]. Another study investigating the awareness levels of professional healthcare workers about CAaN underlines the importance of in-service training on this subject to increase workers’ awareness [34]. According to our research findings, the fact that those who do not know when and how to make a notification constitute 37.4% of the group supports this approach. Informing them all about the entire process from the recognition to the notification of the CAaN is not only important for their own legal responsibilities but also important for being a social problem.

It was observed that participants hesitated to report cases of child abuse and neglect due to different concerns. One of the most important factors is that what needs to be done in the administrative process is not done by the relevant authorities. There is a problem of trust in the authorities. It has been determined that this trust problem is high even in the field of child abuse and education.

Low trust in competent authorities in cases of child abuse and neglect is the most important factor in not obtaining the desired results in the determinations and notifications made. Although the experts spare time and manage the identification and follow-up process, the fact that the authorities do not take the necessary action by the competent authorities will increase the negative effects on the child’s life, negatively affecting their reporting. Although those who receive training on child abuse and neglect are aware of the actions to be taken by competent authorities, not seeing the result causes negative effects on the solution of cases.

The analysis revealed that even those who received training wanted to receive it again. This is the largest piece of evidence that the desire for information on the subject has increased. Both the requests for training and hesitation in reporting to the relevant authorities show that there is a need for clear information on process management in child abuse and neglect. How process management in child abuse and neglect will be carried out by competent authorities and the issue of trust in this process constitute a problem for experts.

Training should be organized to eliminate the participants’ trust problems. Clear information on process management and child protection should be provided in training content. Evidence should be presented that competent authorities manage the processes objectively in cases of child abuse and neglect, and opportunities should be provided for physicians to follow up on their notifications. Physicians should be allowed to have a say in the process management of cases that they have identified by taking time.

As is clearly seen in our findings, the trainings and practices on child neglect and abuse to be provided are expected to:

- Meet the needs of the participants and alleviate their concerns.
- Ensure that the person making the report has mastery of the subject.
- Provide assurance that the competent authorities will implement the necessary practices and sanctions.
- Ensure that the child who has been neglected or abused will have a more accurate life from now on.
- Provide trainings to all health professionals on the subject regardless of their previous education.

## Figures and Tables

**Table 1:** Descriptive characteristics of the participants.

Variable	Groups	*n*	%
Gender	Male	183	36.6
	Female	317	63.4
Marital status	Married	439	87.8
	Single	51	10.2
	Other *	10	2
Having child	Yes	445	89
	No	55	11
Task in the family health unit	Family doctor	249	49.8
	Family health worker **	251	50.2
Profession	General practitioner	223	44.6
	Family medicine specialist	24	4.8
	Specialist in other branches of medicine	2	0.4
	Midwife	189	378
	Nurse	50	10
	Community health technician	5	1
	Emergency medical technician	7	1.4
Educational status	Secondary school	5	1
	High school	65	13
	Associate degree	85	17
	University	319	63.8
	Doctorate-Speciality	26	5.2
Total working time in the profession (year)	1–5	29	5.8
	6–10	56	11.2
	11–15	60	12
	16–20	81	16.2
	21–25	124	24.8
	26–30	114	22.8
	≥31	36	7.2
Working time in family health unit	1–3	81	16.2
	4–6	117	23.4
	≥7	302	60.4

*. Divorced, widowed. **. Midwives, nurses, public health technicians, emergency medical technicians.

**Table 2:** Distribution of participants’ responses to questions about trainings they had and their experiences about CAaN.

Questions		*n*	%
Did you receive training on child neglect and abuse during your eduction?	Yes	269	53.8
	No	231	46.2
Did you receive training on child neglect and abuse while doing your job?	Yes	274	54.8
	No	226	45.2
Do you pay attention to the symptoms of child neglect and abuse in children brought for examination and/or follow-up?	Yes	479	95.8
	No	21	4.2
Did you ever come across to a child neglect and abuse case while working?	Yes	137	27.4
	No	363	72.6
Number of child neglect and abuse cases you have diagnosed in the last 1 year	0	417	83.4
	1	51	10.2
	2	19	3.8
	3	12	2.4
	4	1	0.2
Do you want to participate in a training on child neglect and abuse?	Yes	431	86.2
	No	69	13.8
Would you like to be informed of the results of this research?	It does not matter/I do not care	57	11.4
	Yes	396	79.2
	No	47	9.4

**Table 3:** Distribution scale scores.

Scales	Mean ± SD
Overall Scale	3.77 ± 0.36
PSoCA	4.02 ± 0.42
SoCN	3.87 ± 0.60
BSoCAaN	3.79 ± 0.39
CPPtCAaN	3.50 ± 0.49
CCPtAaN	3.32 ± 0.50
FCiCAaN	3.81 ± 0.58

**Table 4:** Comparison of the mean scores according to sociodemographic characteristics.

No	Groups	Overall Scale	PSoCA	SoCN	BSoCAaN	CPPtCAaN	CCPtAaN	FCiCAaN
		Mean ± SD	Mean ± SD	Mean ± SD	Mean ± SD	Mean ± SD	Mean ± SD	Mean ± SD
1	Male (*n* = 183)	3.81 ± 0.39	4.07 ± 0.44	3.92 ± 0.62	3.80 ± 0.42	3.59 ± 0.48	3.32 ± 0.53	3.84 ± 0.55
	Female (*n* = 317)	3.75 ± 0.33	4.00 ± 0.40	3.85 ± 0.58	3.78 ± 0.37	3.44 ± 0.48	3.31 ± 0.48	3.80 ± 0.59
	Test Result	*p* = 0.065	*p* = 0.071	*p* = 0.196	*p* = 0.624	*p* = 0.001 *	*p* = 0.825	*p* = 0.456
2	Married (*n* = 439)	3.77 ± 0.35	4.02 ± 0.41	3.85 ± 0.59	3.78 ± 0.38	3.49 ± 0.49	3.33 ± 0.49	3.81 ± 0.58
	Single (*n* = 51)	3.81 ± 0.42	4.05 ± 0.49	4.01 ± 0.62	3.87 ± 0.46	3.51 ± 0.51	3.23 ± 0.54	3.80 ± 0.59
	Test Result	*p* = 0.453	*p* = 0.532	*p* = 0.079	*p* = 0.128	*p* = 0.843	*p* = 0.203	*p* = 0.910
3	≥1 child (*n* = 445)	3.76 ± 0.35	4.01 ± 0.42	3.84 ± 0.59	3.78 ± 0.39	3.49 ± 0.49	3.32 ± 0.49	3.80 ± 0.57
	No child (*n* = 55)	3.84 ± 0.37	4.09 ± 0.41	4.11 ± 0.62	3.83 ± 0.40	3.56 ± 0.48	3.28 ± 0.53	3.90 ± 0.59
	Test Result	*p* = 0.116	*p* = 0.219	*p* = 0.004 *	*p* = 0.422	*p* = 0.276	*p* = 0.568	*p* = 0.211
4	<40 (*n* = 157)	3.79 ± 0.33	4.06 ± 0.38	3.96 ± 0.57	3.82 ± 0.36	3.45 ± 0.49	3.32 ± 0.52	3.82 ± 0.59
	40–49 (*n* = 219)	3.75 ± 0.37	3.99 ± 0.43	3.84 ± 0.62	3.75 ± 0.39	3.49 ± 0.48	3.33 ± 0.50	3.79 ± 0.58
	≥50 (*n* = 124)	3.79 ± 0.36	4.03 ± 0.43	3.83 ± 0.59	3.81 ± 0.42	3.57 ± 0.49	3.29 ± 0.48	3.83 ± 0.55
	Test Result	*p* = 0.409	*p* = 0.224	*p* = 0.096	*p* = 0.157	*p* = 0.105	*p* = 0.823	*p* = 0.797
5	≤High school (*n* = 70)	3.71 ± 0.33	3.99 ± 0.36	3.86 ± 0.62	3.75 ± 0.37	3.33 ± 0.50	3.34 ± 0.54	3.67 ± 0.54
	≥Associate (*n* = 430)	3.78 ± 0.36	4.03 ± 0.42	3.87 ± 0.59	3.79 ± 0.39	3.52 ± 0.48	3.31 ± 0.49	3.83 ± 0.58
	Test Result	*p* = 0.106	*p* = 0.439	*p* = 0.877	*p* = 0.389	*p* = 0.002 *	*p* = 0.731	*p* = 0.029 *

SD; standart deviation, * *p* < 0.05; There is a statistically significant difference between the groups.

**Table 5:** Comparison of the mean scores in terms of elements related to working life.

No	Groups	Overall Scale	PSoCA	SoCN	BSoCAaN	CPPtCAaN	CCPtAaN	FCiCAaN
		Mean ± SD	Mean ± SD	Mean ± SD	Mean ± SD	Mean ± SD	Mean ± SD	Mean ± SD
1	FP (*n* = 249)	3.83 ± 0.38	4.07 ± 0.44	3.92 ± 0.60	3.83 ± 0.41	3.62 ± 0.48	3.36 ± 0.49	3.85 ± 0.56
	FHW (*n* = 251)	3.71 ± 0.32	3.97 ± 0.39	3.83 ± 0.59	3.74 ± 0.36	3.37 ± 0.46	3.27 ± 0.50	3.78 ± 0.59
	Test Result	*p* < 0.001 *	*p* = 0.007 *	*p* = 0.081	*p* = 0.013 *	*p* < 0.001 *	*p* = 0.058	*p* = 0.165
2	Yes (*n* = 269)	3.81 ± 0.36	4.06 ± 0.42	3.92 ± 0.63	3.80 ± 0.38	3.54 ± 0.50	3.35 ± 0.51	3.87 ± 0.58
	No (*n* = 231)	3.73 ± 0.35	3.98 ± 0.40	3.82 ± 0.56	3.77 ± 0.39	3.44 ± 0.46	3.28 ± 0.48	3.75 ± 0.56
	Test Result	*p* = 0.012 *	*p* = 0.021 *	*p* = 0.081	*p* = 0.369	*p* = 0.017 *	*p* = 0.099	*p* = 0.026 *
3	Yes (*n* = 274)	3.81 ± 0.36	4.05 ± 0.42	3.90 ± 0.62	3.81 ± 0.39	3.56 ± 0.50	3.34 ± 0.50	3.88 ± 0.58
	No (*n* = 226)	3.72 ± 0.34	3.98 ± 0.40	3.85 ± 0.56	3.75 ± 0.39	3.42 ± 0.45	3.28 ± 0.49	3.73 ± 0.56
	Test Result	*p* = 0.006 *	*p* = 0.066	*p* = 0.353	*p* = 0.075	*p* = 0.001 *	*p* = 0.164	*p* = 0.005 *
4	Yes (*n* = 479)	3.77 ± 0.35	4.02 ± 0.41	3.88 ± 0.60	3.79 ± 0.39	3.50 ± 0.49	3.32 ± 0.50	3.82 ± 0.58
	No (*n* = 21)	3.72 ± 0.38	3.97 ± 0.50	3.79 ± 0.57	3.69 ± 0.43	3.50 ± 0.40	3.33 ± 0.31	3.72 ± 0.54
	Test Result	*p* = 0.493	*p* = 0.600	*p* = 0.525	*p* = 0.260	*p* = 1.000	*p* = 0.865	*p* = 0.466
5	Yes (*n* = 137)	3.80 ± 0.41	4.06 ± 0.46	3.89 ± 0.64	3.80 ± 0.43	3.55 ± 0.53	3.36 ± 0.52	3.82 ± 0.59
	No (*n* = 363)	3.76 ± 0.33	4.01 ± 0.40	3.87 ± 0.58	3.78 ± 0.37	3.48 ± 0.47	3.30 ± 0.49	3.81 ± 0.57
	Test Result	*p* = 0.314	*p* = 0.294	*p* = 0.699	*p* = 0.616	*p* = 0.182	*p* = 0.208	*p* = 0.832
6	Yes (*n* = 431)	3.78 ± 0.35	4.04 ± 0.41	3.88 ± 0.60	3.80 ± 0.39	3.50 ± 0.48	3.32 ± 0.50	3.81 ± 0.58
	No (*n* = 69)	3.71 ± 0.36	3.91 ± 0.41	3.81 ± 0.58	3.69 ± 0.38	3.47 ± 0.50	3.30 ± 0.46	3.82 ± 0.54
	Test Result	*p* = 0.121	*p* = 0.020 *	*p* = 0.357	*p* = 0.030 *	*p* = 0.677	*p* = 0.762	*p* = 0.864
7	≤10 (*n* = 85)	3.82 ± 0.32	4.06 ± 0.38	4.02 ± 0.56	3.84 ± 0.37	3.47 ± 0.49	3.38 ± 0.49	3.89 ± 0.59
	11–20 (*n* = 141)	3.75 ± 0.36	4.03 ± 0.40	3.88 ± 0.63	3.77 ± 0.37	3.44 ± 0.47	3.30 ± 0.50	3.75 ± 0.60
	≥21 (*n* = 274)	3.76 ± 0.36	4.01 ± 0.44	3.82 ± 0.58	3.77 ± 0.40	3.53 ± 0.49	3.30 ± 0.50	3.82 ± 0.56
	Test Result	*p* = 0.372	*p* = 0.610	*p* = 0.031 *	*p* = 0.363	*p* = 0.224	*p* = 0.448	*p* = 0.218

FP: Family physician, FHW: Family health worker (midwife, nurse, community health technician, emergency medical technician), SD; standart deviation, * *p* < 0.05; There is a statistically significant difference between the groups.

**Table 6:** Distribution of the reason why physicians who observe evidence of child abuse and/or neglect do not report this to the relevant authorities according to variables.

Variable			The Reason Why Physicians Who Observe Evidence of Child Abuse and/or Neglect Do Not Report This to the Relevant Authorities *					Total
			Thinking That the Relevant Authorities Will Not Take the Necessary Actions	A Desire Not to Get Involved in an Unpleasant Topic	Not Knowing When to Notify or How to Notify	It Is Thought That the Life of the Child in Question Will Be Negatively Affected After the Notification	Not Suspecting Abuse and Neglect	
Gender	M	*n*	74	76	60	62	59	161
		%	46.0	47.2	37.3	38.5	36.6	
	F	*n*	136	100	127	120	83	284
		%	47.9	35.2	44.7	42.3	29.2	
Having child status	Y	*n*	182	156	162	162	124	392
		%	46.4	39.8	41.3	41.3	31.6	
	N	*n*	28	20	25	20	18	53
		%	52.8	37.7	47.2	37.7	34.0	
Did you receive training on child neglect and abuse during your eduction?	Y	*n*	118	86	95	92	74	234
		%	50.4	36.8	40.6	39.3	31.6	
	N	*n*	92	90	92	90	68	211
		%	43.6	42.7	43.6	42.7	32.2	
Did you receive training on child neglect and abuse while doing your job?	Y	*n*	122	97	101	104	74	239
		%	51.0	40.6	42.3	43.5	31.0	
	N	*n*	88	79	86	78	68	206
		%	42.7	38.3	41.7	37.9	33.0	
Do you pay attention to the findings of child neglect and abuse in children brought for examination and follow-up?	Y	*n*	204	167	177	174	132	425
		%	48.0	39.3	41.6	40.9	31.1	
	N	*n*	6	9	10	8	10	20
		%	30.0	45.0	50.0	40.0	50.0	
Do you want to participate in a training on child neglect and abuse?	Y	*n*	179	154	168	158	125	383
		%	46.7	40.2	43.9	41.3	32.6	
	N	*n*	31	22	19	24	17	62
		%	50.0	35.5	30.6	38.7	27.4	
Total		*n*	210	176	187	182	142	445

M; Male, F; Female, Y; Yes, N; No, * The sum of the row percentages may exceed 100% due to multiple answers to the question.

**Table 7:** Distribution of the topics to be covered in a training on child abuse and neglect according to variables.

Variable			Education Subject *				Total
			Information About the Signs and Symptoms of Child Abuse and Neglect	Information on What Can Be Done in Case of Detection of Child Abuse and/or Neglect	Information on Legal Regulations Regarding the Reporting of Child Abuse and/or Neglect	Information on the Experiences of Children Who Have Been Reported to the Authorities for Abuse and/or Neglect After This Process	
Gender	M	*n*	126	149	130	124	174
		%	72.4	85.6	74.7	71.3	
	F	*n*	215	245	211	224	296
		%	72.6	82.8	71.3	75.7	
Having child status	Y	*n*	299	351	305	306	417
		%	71.7	84.2	73.1	73.4	
	N	*n*	42	43	36	42	53
		%	79.2	81.1	67.9	79.2	
Did you receive training on child neglect and abuse during your eduction?	Y	*n*	182	208	193	189	251
		%	72.5	82.9	76.9	75.3	
	N	*n*	159	186	148	159	219
		%	72.6	84.9	67.6	72.6	
Did you receive training on child neglect and abuse while doing your job?	Y	*n*	174	208	186	194	256
		%	68.0	81.3	72.7	75.8	
	N	*n*	167	186	155	154	214
		%	78.0	86.9	72.4	72.0	
Do you pay attention to the findings of child neglect and abuse in children brought for examination and follow-up?	Y	*n*	326	378	327	336	452
		%	72.1	83.6	72.3	74.3	
	N	*n*	15	16	14	12	18
		%	83.3	88.9	77.8	66.7	
Do you want to participate in a training on child neglect and abuse?	Y	*n*	316	363	316	318	420
		%	75.2	86.4	75.2	75.7	
	N	*n*	25	31	25	30	50
		%	50.0	62.0	50.0	60.0	
Total		*n*	341	394	341	348	470 **

M; Male, F; Female, Y; Yes, N; No, * The sum of the row percentages may exceed 100% due to multiple answers to the question. ** 470 participants answered the question.

**Table 8:** Multiple Regression Analysis for The Diagnosis Scale of The Risks and Symptoms of Child Abuse and Neglect.

Dependent Variable	Independent Variable	Model Statistics			Coefficent Statistics			
		R^2^	F	*p* ^1^	β_1_	β_2_	t	*p* ^2^
Overall Score	(Constant)	0.046	8.963	0.001 *	4.228		20.597	0.001 *
	Age				−0.028	−0.059	−0.827	0.409
	Gender				0.020	0.027	0.431	0.667
	Marital status				0.028	0.031	0.612	0.541
	Having child status				0.049	0.042	0.809	0.419
	Task in the family health unit				−0.147	−0.204	−4.428	0.001 *
	Education				0.024	0.048	0.857	0.392
	Total working time in the profession (year)				−0.047	−0.099	−2.165	0.031 *
	Receive training on child neglect and abuse during eduction				−0.089	−0.123	−2.802	0.005 *
	Receive training on child neglect and abuse while doing job?				−0.059	−0.081	−1.570	0.117
	Do you pay attention to the symptoms of child neglect and abuse in children brought for examination and/or follow-up?				−0.052	−0.029	−0.649	0.516
	Did you ever come across to a child neglect and abuse case while working?				−0.008	−0.010	−0.224	0.823
	Do you want to participate in a training on child neglect and abuse?				−0.076	−0.073	−1.614	0.107

β_1_; Unstandardized regression coefficients, β_2_; Standardized regression coefficients, *p* ^1^; significance value of the model, * *p* ^2^ < 0,05; *t*-test result for the significance of the regression coefficients, R^2^; Determination coeffient.

## Data Availability

Research data is confidential and will not be published.
